# Cognitive Decline in Hemodialysis Patients: A Cross-Sectional Study Using Addenbrooke's Cognitive Examination-III (ACE-III)

**DOI:** 10.7759/cureus.92326

**Published:** 2025-09-14

**Authors:** Siri Chandana, Jayakumar M, Sandhya Suresh, Manikantan Sekhar

**Affiliations:** 1 Department of Nephrology, Sri Ramachandra Institute of Higher Education and Research, Chennai, IND

**Keywords:** ace-iii, addenbrooke's cognitive examination-iii (ace-iii), chronic kidney disease, cognitive impairment, haemodialysis, india, neurocognition

## Abstract

Background

Among patients with chronic kidney disease (CKD) undergoing maintenance hemodialysis (HD), cognitive impairment (CI) is a significant yet often unknown side effect. This study used Addenbrooke's Cognitive Examination-III (ACE-III) to assess the prevalence and pattern of cognitive dysfunction in a South Indian HD population. It also aimed to determine the correlations with clinical and sociodemographic factors.

Methods

A cross-sectional study was conducted in a tertiary care facility in Chennai, Tamil Nadu, India, involving 175 patients undergoing maintenance HD for more than three months. The Indian English version of the ACE-III was used to evaluate cognitive function. Clinical and demographic data, including age, gender, education, duration of dialysis, comorbidities, and hemoglobin levels, were collected and analyzed. A p-value of <0.05 was considered statistically significant.

Results

Almost half of the patients, that is, 82 (46.9%), were classified as having CI with ACE-III scores of 60-80. Lower cognitive scores were significantly associated with greater age, lower educational levels, and longer time spent on dialysis (p<0.05).

Conclusion

In South India, CI is prevalent among HD patients, with memory and attention being the areas that are most affected. Age, level of education, and dialysis duration are contributing factors. In dialysis care, routine ACE-III screening can facilitate early detection and targeted interventions.

## Introduction

Over 800 million people worldwide suffer from chronic kidney disease (CKD), which is linked to high rates of morbidity, mortality, and medical costs [1]. Hemodialysis (HD), the most popular renal replacement therapy for end-stage renal disease (ESRD), is life-sustaining, but it also induces physiological stress that can result in neurological problems like cognitive impairment (CI) [2]. Up to 70% of dialysis patients experience CI, which is associated with mortality, lowers quality of life, and causes poor treatment adherence [3,4]. Cognitive screening is still underutilized in HD care despite its burden, particularly in limited resource settings such as India [5]. The accumulation of neurotoxins, vascular damage, inflammation, and cerebral hypoperfusion during dialysis are some of the mechanisms that underlie CI in HD [6]. In HD patients, brain imaging has revealed cortical thinning, white matter lesions, and hippocampal atrophy [7]. Among the factors influencing cognitive outcomes are age, anemia, dialysis duration, and educational attainment [8]. The objectives of the present study were to use the ACE-III to measure cognitive function in HD patients and evaluate the clinical and demographic predictors of CI.

## Materials and methods

Study design and population

A cross-sectional study was conducted from May to September 2024 in the Department of Nephrology, Sri Ramachandra Institute of Higher Education and Research, Chennai, Tamil Nadu, India. The study protocol was approved by the Institutional Ethics Committee, and written informed consent was obtained from all participants. The study was conducted in accordance with the Declaration of Helsinki.

The study included adult patients (≥18 years) with ESRD receiving maintenance HD for at least three months. Patients were excluded if they had acute medical instability, pre-existing psychiatric or neurological disorders, documented CI, active substance abuse, or significant visual/hearing impairments that could interfere with testing.

Data collection

A structured questionnaire was used to collect demographic data including age, gender, educational level (categorized as primary education or less, secondary education, and higher education), and occupation. Clinical data collected included primary cause of CKD, dialysis vintage (duration since initiation of HD in months), dialysis frequency, vascular access type, and comorbid conditions such as diabetes mellitus and hypertension.

Cognitive assessment

Addenbrooke's Cognitive Examination-III (ACE-III) Indian English version was used for cognitive assessment [9]. This validated instrument evaluates five cognitive domains with a maximum total score of 100 points: attention and concentration (18 points), memory (26 points), fluency (14 points), language (26 points), and visuospatial skills (16 points). CI was defined as a total ACE-III score of ≤82 points, with severity classified as mild (69-82 points), moderate (60-68 points), or severe (<60 points).

Statistical analysis

Statistical analysis was performed using SPSS Version 20.0 (IBM Corp., Armonk, NY). Continuous variables were expressed as mean ± standard deviation or median with interquartile range. Categorical variables were presented as frequencies and percentages. The chi-square test and ANOVA were used to examine associations between categorical variables and CI status. A p-value of <0.05 was considered statistically significant for all analyses.

## Results

Table 1 depicts the patient demographic details. Among 175 cases, 56 (32%) were female and 119 (68%) were male. The mean age was 56.3 ± 11.7 years. Regarding educational attainment, patients were distributed across various levels, with the largest group having completed 12th grade or less (40.0%, n=70), followed by college graduates with BA (Bachelor of Arts) or BS (Bachelor of Science) degrees (25.1%, n=44), and high school graduates or GED (general educational development) holders (23.4%, n=41). A smaller proportion had graduate degrees such as MD (Doctor of Medicine), PhD (Doctor of Philosophy), or JD (juris doctor) (9.7%, n=17), while only 1.7% (n=3) reported some college or technical training. In terms of dialysis vintage, the majority of participants were relatively new to dialysis treatment, with 66.3% (n=116) having 0-24 months of experience, 28.6% (n=50) having 25-72 months of experience, and only 5.1% (n=9) having more than 72 months of dialysis experience.

**Table 1:** Demographic characteristics of study participants Data are presented as n (%). BA, Bachelor of Arts; BS, Bachelor of Science; GED, general educational development; MD, Doctor of Medicine; PhD, Doctor of Philosophy; JD, juris doctor

Variable	Frequency (n=175)	Percent (%)
Sex		
Male	119	68.0
Female	56	32.0
Education		
12th grade or less	70	40.0
High school graduate or GED	41	23.4
Some college/technical training	3	1.7
College graduate (BA or BS)	44	25.1
Graduate degree (MD, PhD, JD)	17	9.7
Dialysis vintage		
0–24 months	116	66.3
25–72 months	50	28.6
>72 months	9	5.1

Table 2 illustrates the distribution of ACE-III scores among the patient population. The mean ACE-III score was 78.4 ± 11.3. There were 128 (73.2%) patients with CI (≤82), 82 (46.9%) patients with moderate impairment, and 46 (26.3%) patients with mild impairment.

**Table 2:** Distribution of total ACE-III scores Data are presented as n (%). ACE-III, Addenbrooke's Cognitive Examination-III

Score Range	Frequency	Percent	Cumulative Percent
60–80	82	46.9%	46.9%
81–90	46	26.3%	73.1%
91–100	47	26.9%	100.0%

Table 3 shows the cognitive domain scores as per ACE-III subscales, among the patient population. The cognitive domain assessment among 175 patients showed varying performance patterns. In attention, 62.3% (n=109) scored 7-12, while 37.7% (n=66) scored 13-18. Memory performance was more evenly distributed, with 44.0% (n=77) scoring 10-18 and 56.0% (n=98) scoring 19-26. Most patients demonstrated strong fluency abilities, with 77.7% (n=136) scoring 11-14 compared to 22.3% (n=39) scoring 6-10. Language performance was similarly favorable, with 76.0% (n=133) achieving scores of 19-26 and 24.0% (n=42) scoring 10-18. Visuospatial function showed the strongest performance overall, with 94.3% (n=165) scoring 11-16 and only 5.7% (n=10) scoring 6-10.

**Table 3:** Cognitive domain scores (ACE-III subscales) Data are presented as n (%). ACE-III, Addenbrooke's Cognitive Examination-III

Domain	Frequency	Percent (%)
Attention		
7–12	109	62.3
13–18	66	37.7
Memory		
10–18	77	44.0
19–26	98	56.0
Fluency		
6–10	39	22.3
11–14	136	77.7
Language		
10–18	42	24.0
19–26	133	76.0
Visuospatial		
6–10	10	5.7
11–16	165	94.3

Chi-square analyses revealed significant associations between CI status and key demographic and clinical variables (Table 4). Educational level demonstrated the strongest association with CI (χ² = 22.5, df = 4, p < 0.001, Cramér's V = 0.36), indicating a medium-to-large effect size. Patients with lower educational attainment showed progressively higher rates of CI, ranging from 58.8% in those with graduate degrees to 82.9% in those with 12th-grade education or less. Age was also significantly associated with CI (χ² = 13.5, df = 1, p < 0.001, Cramér's V = 0.28), with patients aged ≥60 years showing substantially higher impairment rates (82.9%) compared to younger patients (66.7%). While dialysis vintage showed a trend toward increased CI with longer duration (88.9% in patients on dialysis for more than 72 months vs 69.8% in those on dialysis for less than 24 months), this association did not reach statistical significance (χ² = 2.38, df = 2, p > 0.05). Similarly, hemoglobin levels below 10 g/dL were associated with higher CI rates (77.6% vs. 68.9%), but this relationship was marginally non-significant (χ² = 3.08, df = 1, p = 0.08). Sex showed no meaningful association with CI status (χ² = 0.0005, df = 1, p > 0.05, Cramér's V = 0.002).

**Table 4:** Chi-square test statistics for associations with cognitive impairment status *Indicates statistically significant result at the p<0.001 level. BA, bachelor of arts; BS, bachelor of science; GED, general educational development; MD, doctor of medicine; PhD, doctor of philosophy; JD, juris doctor

Variable	Normal Cognition, n (%)	Cognitive Impairment, n (%)	Total, N	χ² value	df	p-Value	Cramér's V
Gender							
Male	32 (27.7)	87 (73.1)	119	0.0005	1	>0.05	0.002
Female	15 (26.8)	41 (73.2)	56				
Education level							
12th grade or less	12 (17.1)	58 (82.9)	70	22.5*	4	<0.001	0.36
High school graduate/GED	10 (24.4)	31 (75.6)	41				
Some college/technical	1 (33.3)	2 (66.7)	3				
College graduate (BA/BS)	17 (38.6)	27 (61.4)	44				
Graduate degree (MD/PhD/JD)	7 (41.2)	10 (58.8)	17				
Dialysis vintage							
0-24 months	35 (30.2)	81 (69.8)	116	2.38	2	>0.05	0.12
25-72 months	11 (22.0)	39 (78.0)	50				
>72 months	1 (11.1)	8 (88.9)	9				
Age group							
<60 years	35 (33.3)	70 (66.7)	105	13.5*	1	<0.001	0.28
≥60 years	12 (17.1)	58 (82.9)	70				
Hemoglobin level							
≥10 g/dL	28 (31.1)	62 (68.9)	90	3.08	1	0.08	0.13
<10 g/dL	19 (22.4)	66 (77.6)	85				

The overall and domain-specific ACE-III scores were significantly lower in patients aged ≥60 years (p<0.001). There was a positive correlation (p<0.001) between cognitive scores and educational level. Dialysis duration longer than six years was significantly associated with lower ACE-III scores (p=0.001). Lower scores were linked to hemoglobin levels below 10 g/dL, although this association was not statistically significant (p=0.08).

## Discussion

This study adds to the increasing amount of data, indicating that among patients receiving maintenance HD, CI is a common and underreported complication. According to our findings, the most common presentation of CI was moderate, with nearly three-fourths of study participants having some kind of CI. These results corroborate previous research that indicated a 30% to 70% prevalence of CI in HD populations worldwide [3,4,6,10]. They also show the universal impact of renal insufficiency and its treatment modalities on brain function.

The use of ACE-III allowed for a thorough comprehension of several cognitive domains. In a meta-analysis of almost 50 studies, the prevalence of CI assessed using the ACE-III was 40%, reflecting a substantial burden of cognitive dysfunction among CKD patients [11].

Attention and memory were the most affected among these, based on damage to frontal-subcortical circuits and hippocampus structures, which are particularly susceptible to the fluctuating hemodynamic environment of HD and the chronic metabolic insults of CKD [6,7]. The relatively intact language and visuospatial abilities suggest a non-Alzheimer's profile more consistent with subcortical vascular CI.

Age and cognitive decline

Our cohort's ACE-III scores were substantially lower for those over 60. This result is consistent with the well-established link between aging and cognitive decline, which is made worse in patients with CKD by endothelial dysfunction, cerebrovascular pathology, and cumulative oxidative stress [8]. According to Zheng et al. [7], elderly patients may also be more neuronally vulnerable to dialysis-induced hypotension, which, over time, may result in cerebral hypoperfusion and structural alterations in the brain.

Education and cognitive reserve

There was a significant protective effect of educational attainment. Cases with higher-education levels performed better in all cognitive domains. This supports the credence to the cognitive reserve hypothesis, which holds that people who have engaged in more intellectual activities throughout their lives are better able to tolerate neuropathological damage before symptoms appear [12]. The frequent metabolic stressors associated with long-term HD treatment may be mitigated by cognitive reserve.

Dialysis vintage and cognitive function

The length of dialysis was found to be a significant predictor of CI. ACE-III scores were significantly lower for patients who had been receiving dialysis for more than six years. According to research, chronic HD causes frequent episodes of cerebral hypoperfusion, which, over time, can cause microvascular damage, atrophy, and silent infarcts [13].

Moreover, the chronic inflammatory state of ESRD may intensify these effects because pro-inflammatory cytokines such as TNF-alpha and IL-6 have been connected to neurodegeneration [14].

Anemia and cognitive outcomes

A trend toward poorer cognitive performance was noted in our study, even though anemia (Hb <10 g/dL) did not reach statistical significance. One frequent side effect of CKD is anemia, which lowers the amount of oxygen that reaches the brain's tissues. Anemia and CI, specifically in attention and processing speed, have been linked in previous research [15]. It is possible that our study lacked the power to identify this relationship conclusively; therefore, further investigation is necessary.

Clinical and public health implications

Routine cognitive screening should be incorporated into dialysis care due to the high prevalence of CI and its clinical effects in HD patients, which include poor treatment adherence, increased hospitalization, and decreased quality of life. Tools such as the ACE-III are helpful, reasonably quick, and provide domain-specific data that can guide customized interventions. The identification of at-risk individuals enables proactive measures such as medication simplification, cognitive ability-based dietary counselling, and increased caregiver involvement.

In low- and middle-income nations like India, the burden of CI may be exacerbated by delayed CKD diagnosis, limited access to cognitive rehabilitation, and low health literacy. Multidisciplinary care models involving nephrologists, neurologists, psychologists, and social workers are desperately needed. Teaching caregivers and dialysis staff about the early signs of cognitive decline can improve outcomes.

Longitudinal studies are also needed to assess the effectiveness of interventions such as erythropoiesis-stimulating agents, intradialytic cognitive training, or innovative dialysis protocols intended to reduce cerebral ischemia and ascertain whether CI worsens over time in dialysis patients.

Limitations

There are several limitations to this study. First, it is impossible to evaluate causality or CI over time due to the study’s cross-sectional design. Second, cultural and educational factors may still influence the ACE-III even after it has been validated. Third, the single-center design may limit generalizability to larger populations.

## Conclusions

This study helps raise awareness of CI in HD populations in India and globally. The findings are in favor of routine cognitive screening, especially for patients who have been on dialysis for a long time, are elderly, or have low educational attainment. In order to improve patient outcomes and quality of life, cognitive health must be addressed in nephrology care.
